# Patient–Spouse Experiences of Post‐Stroke Life Reconstruction: A Longitudinal Dyadic Journey Mapping Study

**DOI:** 10.1002/brb3.71914

**Published:** 2026-11-09

**Authors:** Wenbo Li, Qingfang Ye, Haiyang Zhang, Meng Wang, Yuying Fan, Qiujie Li

**Affiliations:** ^1^ Department of Clinical Nursing Education The Second Affiliated Hospital of Harbin Medical University Harbin China; ^2^ Department of Nursing The Harbin Medical University Harbin China; ^3^ Department of Urology The Third Affiliated Hospital of Harbin Medical University Harbin China; ^4^ Department of Basic Medical Sciences Hebei Medical University Shijiazhuang China

**Keywords:** dyadic illness management, limb motor impairment, longitudinal qualitative research, patient journey mapping, spousal caregiver, stroke

## Abstract

**Background:**

Young and middle‐aged stroke survivors with residual limb motor impairment continue rehabilitation while resuming employment, family responsibilities, and social roles. Their spouses often provide care and rehabilitation support, yet little is known about how partners’ experiences compare and change within dyads over time. This study aimed to examine the experiences of young and middle‐aged stroke survivors with limb motor impairment and their spouses across inpatient recovery, early transition home, and longer‐term home‐based life reconstruction, and to synthesize the findings into a patient–spouse dual‐line journey map.

**Methods:**

This longitudinal qualitative study recruited 18 patient–spouse dyads using maximum‐variation purposive sampling. Patients and spouses were interviewed separately 1–3 days before discharge (T1), 4 weeks after discharge (T2), and 6 months after discharge (T3), yielding 108 interviews. Data were analyzed using the Framework Method adapted for longitudinal and dyadic qualitative data. Longitudinal matrices and within‐ and between‐dyad comparisons informed the visual synthesis of findings.

**Results:**

Findings were synthesized across three phases: T1: Inpatient recovery and preparation for discharge; T2: Early transition home and emerging differences in independence and safety; and T3: Longer‐term life reconstruction and varied dyadic adjustment. Five cross‐cutting analytic domains informed this synthesis: patients’ rehabilitation and everyday‐life experiences; spouses’ caregiving experiences; convergence and difference within dyads; emotional experiences; and stage‐specific support needs and service gaps. At T1, professional guidance provided reassurance, while preparation for home remained incomplete. At T2, patients’ attempts at independence coexisted with spouses’ safety concerns and difficulties applying discharge guidance. At T3, negotiated goals and reassurance in some dyads contrasted with restricted participation, caregiving strain, or reduced communication in others. These findings informed a patient–spouse dual‐line journey map.

**Conclusions:**

Patients’ rehabilitation and everyday‐life experiences and spouses’ caregiving experiences changed across the hospital‐to‐home transition, with differing priorities within dyads and varied longer‐term adjustment. Findings support involving both partners in discharge preparation and providing individualized follow‐up, rehabilitation, and caregiver support responsive to changing needs.

## Graphical abstract

In 18 patient–spouse dyads, experiences shifted from shared reliance on clinicians before discharge to differing views of independence and safety at home. By six months, adjustment varied; findings support joint discharge preparation, individualized follow‐up, and ongoing assessment of rehabilitation and caregiver needs.

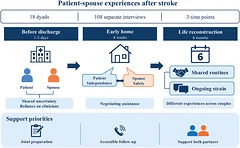

**Abbreviations**
COREQ: Consolidated Criteria for Reporting Qualitative Research
P: patient
S: spouse
T1: first interview conducted 1–3 days before discharge
T2: second interview conducted 4 weeks after discharge
T3: third interview conducted 6 months after discharge.

## 1 Introduction

Stroke is a leading cause of long‐term disability and imposes substantial demands on survivors, families, and healthcare systems (Langhorne et al. 2011). After acute treatment, many survivors return home with persistent rehabilitation needs and dependence in daily activities (Chen, Xiao, Chamberlain, and Newman 2021; Oppici et al. 2026). Families may struggle to apply discharge information, access rehabilitation, and obtain timely professional support (O'Callaghan et al. 2024). For young and middle‐aged survivors, recovery also intersects with employment, parenting, household responsibilities, and social participation (Cleary et al. 2025; Liu et al. 2025). Spouses commonly become the principal providers of practical help, rehabilitation support, safety monitoring, and emotional support while maintaining their own work and family responsibilities (Song et al. 2025). Rehabilitation is therefore a shared process in which both partners adapt to changing physical, emotional, and practical demands.

Qualitative studies have described changing rehabilitation, caregiving, emotional, and support needs during the hospital‐to‐home transition (Chen et al. 2022; Chen et al. 2021), and research increasingly addresses shared stroke management within patient–caregiver dyads (Song et al. 2025; Wang, Pucciarelli, Mei, Zhang, and Vellone 2023). Longitudinal evidence also shows that both perspectives change over time (Lin et al. 2022). However, group‐level analyses provide limited insight into how experiences are negotiated within individual couples or how convergence and divergence evolve across recovery. Young and middle‐aged survivors with residual limb motor impairment are particularly underrepresented.

The Theory of Dyadic Illness Management provided the relational framework for this study. It sensitized the research team to examine how patients and spouses appraised stroke‐related demands, responded to rehabilitation and caregiving challenges, communicated about illness management, and negotiated responsibilities within the dyad. The theory also guided attention to both convergence and divergence between partners, while allowing the analysis to identify experiences that extended beyond the framework (Lyons and Lee 2018).

Journey mapping organizes experiences, actions, emotions, service encounters, and unmet needs over time (Saragosa et al. 2023; Trebble et al. 2010), and has identified stage‐specific rehabilitation needs (Yuan et al. 2026). Conventional maps, however, usually privilege the patient perspective. A dual‐line map can present patient and spouse experiences in parallel, although it should communicate qualitative findings rather than imply a universal or causal recovery pathway.

This longitudinal qualitative study explored how young and middle‐aged stroke survivors with residual limb motor impairment and their spouses experienced rehabilitation, everyday life, caregiving, communication and shared decision‐making, emotional adjustment, and support needs during inpatient recovery, the transition home, and longer‐term life reconstruction. It also examined how these experiences converged or diverged within dyads over time and synthesized the findings into a patient–spouse dual‐line journey map highlighting phase‐specific challenges and support priorities.

## 2 Methods

### 2.1 Study Design

This study adopted an interpretivist qualitative orientation, assuming that patients’ and spouses’ experiences of stroke rehabilitation and family life are context‐dependent and shaped through personal interpretation and interaction within the dyad. Consistent with this orientation, the study used a primarily inductive analytic approach, while drawing sensitively on the Theory of Dyadic Illness Management to attend to relational processes, including partners’ illness appraisals, management behaviors, and patterns of dyadic interaction (Lyons and Lee 2018). The study employed a longitudinal qualitative design to examine how these experiences and relational processes changed over time. It was conducted in the Department of Rehabilitation at the Second Affiliated Hospital of Harbin Medical University, China. From May 2025 to March 2026, patients and spouses were interviewed separately 1–3 days before discharge (T1), 4 weeks after discharge (T2), and 6 months after discharge (T3), capturing discharge preparation, early home adjustment, and longer‐term rehabilitation. Reporting followed the Consolidated Criteria for Reporting Qualitative Research (COREQ) (Supplementary File 1) (Tong, Sainsbury, and Craig 2007).

### 2.2 Participants and Recruitment

Maximum‐variation purposive sampling was operationalized using a participant‐characteristics questionnaire to monitor variation in prespecified demographic, clinical, and caregiving characteristics. These included patients’ sex, age, educational attainment, employment status, place of residence, stroke type, first‐ever or recurrent stroke, and side of limb motor impairment, as well as spouses’ sex, age, educational attainment, employment status, self‐reported health status, average daily caregiving time, and previous caregiving experience. Clinical staff identified potentially eligible inpatients, after which WL confirmed eligibility and reviewed the characteristics of eligible dyads to seek variation across these dimensions during recruitment. This approach aimed to capture diverse patient–spouse experiences rather than achieve statistical representativeness. Written and verbal information was provided, and written informed consent was obtained from both partners.

Patients were eligible if they: (1) were aged 18–59 years; (2) had a diagnosis of ischemic or hemorrhagic stroke confirmed by computed tomography or magnetic resonance imaging and a specialist physician; (3) had unilateral or bilateral limb motor impairment during hospitalization that was expected to persist until discharge and required assistance or safety supervision in at least some activities of daily living; (4) were married, with their spouse acting as their primary family caregiver; (5) were conscious and able to participate in an interview in Mandarin Chinese; and (6) voluntarily participated and provided written informed consent. Patients were excluded if they: (1) had a documented severe cognitive impairment or mental disorder that prevented meaningful participation in an interview; or (2) had a serious coexisting condition, including advanced heart, liver, or kidney failure or malignant disease, that substantially affected their ability to participate. Spouses were eligible if they: (1) were the spouse and primary family caregiver of an enrolled patient; (2) were able to participate in an interview in Mandarin Chinese; and (3) voluntarily participated and provided written informed consent. Spouses were excluded if a cognitive, mental, or physical health condition prevented them from participating in an interview or undertaking the primary caregiving role.

Recruitment, data collection, and preliminary analysis proceeded concurrently. Information power was judged from the focused aim, specific dyadic sample, repeated interviews, and depth and variation of data (Malterud et al. 2016). Recruitment ended when sufficient depth and diversity had been achieved and additional dyads were unlikely to add substantively different information.

### 2.3 Research Team and Reflexivity

The six‐member team had nursing, nursing‐education, and medical backgrounds. WL, a doctoral nursing student trained in qualitative interviewing and reflexivity, recruited participants, conducted interviews, managed data, and coded initially. QY, HZ, and MW reviewed the framework, extracts, matrices, and interpretations; YF and QL supervised the methods and journey map and helped resolve disagreements. WL had clinical rehabilitation nursing contact with potential participants but did not make their treatment decisions. Participants were informed of WL's role and the study purpose. Reflexive memos documented assumptions, responses, interpretations, and possible clinical‐background effects.

### 2.4 Data‐Collection Instruments

#### 2.4.1 Participant Characteristics Questionnaire

A researcher‐developed questionnaire was used to collect relevant demographic, clinical, and caregiving information. Patient information included sex, age, educational attainment, employment status, place of residence, stroke type, first‐ever or recurrent stroke, and site of limb motor impairment at discharge. Spouse information included sex, age, educational attainment, employment status, self‐reported health status, average daily caregiving time, and previous caregiving experience.

#### 2.4.2 Longitudinal Semi‐Structured Interview Guide

The corresponding patient and spouse interview guides were informed by the Theory of Dyadic Illness Management and addressed illness appraisals, rehabilitation and daily activities, caregiving, dyadic communication and decision‐making, emotional responses, services, and unmet needs. Questions were designed to explore not only everyone's experience but also how partners understood one another's concerns, negotiated assistance and independence, and managed disagreements about risk and caregiving. T1 focused on hospitalization and discharge; T2 on early home experiences; and T3 on longer‐term rehabilitation, role adjustment, and support. The theory informed the areas of inquiry but did not restrict participants’ responses or predetermine the analytic categories or findings (Supplementary File 2).

### 2.5 Data Collection

WL conducted T1 interviews face‐to‐face in a private hospital room and T2–T3 interviews in private participant‐selected settings, documenting mode and location. Partners were interviewed separately without the other partner present and were told that individual disclosures would not be shared. This approach was chosen to protect privacy and reduce the pressure to present a shared or socially acceptable account in front of one another, thereby allowing patients and spouses to discuss sensitive issues, including emotional distress, caregiving burden, disagreements about independence and safety, and unmet needs. Separate interviews also enabled an independent comparison of each partner's perspective within the dyad over time. Mandarin interviews followed flexible guides, with probing, clarification, and end‐of‐interview summaries for correction or addition. Interviews lasted 30–60 min and were audio‐recorded; field notes and reflexive memos documented context and follow‐up issues. WL transcribed recordings verbatim within 24 h, checked them, and removed identifiers. Corresponding codes (e.g., P01 and S01) linked dyad members. WL translated selected quotations into English; QY checked them against the Chinese originals, and disagreements were resolved with YF, prioritizing intended meaning.

### 2.6 Data Analysis

The analysis was informed by an interpretivist qualitative orientation, which views participants’ experiences as contextually situated and shaped through personal meaning‐making and interaction (Denzin and Lincoln 2017). We adopted an inductive–deductive analytic approach. The Framework Method was adapted for longitudinal dyadic data (Collaço et al. 2021; Gale et al. 2013). The Theory of Dyadic Illness Management was used as a sensitizing framework rather than a predetermined coding scheme (Lyons and Lee 2018). It guided attention to partners’ appraisals of illness‐related demands and risks, their individual and shared management behaviors, and the ways in which responsibilities and decisions were negotiated. These concepts were examined longitudinally and comparatively across patients and spouses, while inductive coding allowed unanticipated issues to emerge.

Analysis proceeded through familiarization, initial coding, development and refinement of the analytical framework, charting, and interpretation. First, WL read each transcript alongside the field notes and reflexive memos and coded the data primarily inductively. Codes with related meanings were grouped into provisional analytical categories and broader domains. The emerging codes were then considered in relation to the theoretical concepts, while data that did not fit the framework were retained. QY, HZ, and MW reviewed selected coded extracts across participants, perspectives, and time points. Through team discussion, the code definitions, analytical categories, domains, and analytical framework were refined, after which WL applied the revised framework to the full dataset.

The adaptation of the Framework Method involved separate and paired charting procedures. Longitudinal matrices were constructed for each participant to compare themes and contextual information across T1–T3. Patient and spouse accounts were initially retained as distinct perspectives to preserve the specificity of each partner's experience. Dyadic comparison was then conducted by examining paired extracts from the same dyad and time point, allowing convergence, divergence, and complementarity to be identified and interpreted in relation to functional impairment, family roles, caregiving demands, access to professional support, and changes in care settings. Between‐dyad comparisons further explored how these contextual factors contributed to variation across couples. YF and QL reviewed the links among the dyadic data, contextual information, interpretations, and illustrative quotations. Disagreements were resolved through discussion and reconsideration of the original transcripts. Numerical intercoder agreement was not calculated because team review aimed to refine interpretation rather than establish statistical reliability.

Five analytic domains were used as broad organizing categories for comparisons across patients, spouses, and interview periods. We use the term “domains” rather than “themes” to emphasize that these categories group related aspects of experience, while more specific patterns of meaning were interpreted within and across them. Findings across the domains were integrated within each interview period to examine the relationships among rehabilitation, caregiving, dyadic interaction, emotional experiences, and support needs. The phase boundaries followed the interview schedule, while the descriptive phase titles summarized the integrated findings within each period. Chronological presentation was chosen to retain the relationships among these experiences as the care context changed, while comparisons within and between dyads preserved variation in how partners experienced each phase.

### 2.7 Construction of the Dual‐Line Journey Map

The dual‐line journey map was constructed with the five analytic domains arranged as rows and the three interview periods arranged as columns. The column headings combine each interview period with its integrated phase‐related finding and reproduce the corresponding Results headings verbatim. Within each phase, the map presents patient and spouse experiences in parallel, alongside within‐dyad convergence and difference, emotional experiences, and support needs. The map is a visual synthesis of the interview findings rather than an independent data source. The full team reviewed drafts, and two stroke‐rehabilitation specialists and two qualitative researchers reviewed their clarity, clinical relevance, and consistency with the findings. Changes were made only when supported by the data.

### 2.8 Rigor

Credibility was supported by repeated separate interviews, clarification and summary, transcript comparison, contrasting‐case analysis, and multi‐researcher review. At the end of each interview, the interviewer briefly summarized the participant's own account and invited the participant to correct, clarify, or add information. These summaries concerned only the individual participant's interview and were not shared with the spouse or other participants. This process was used to confirm the accuracy of the interviewer's immediate understanding and to guide subsequent interviews; it was not formal member checking of the completed thematic findings. An audit trail supported dependability; reflexive memos and alternative‐interpretation discussions supported confirmability; and detailed contextual and participant information supported transferability.

### 2.9 Ethical Considerations

The Ethics Committee of the Second Affiliated Hospital of Harbin Medical University approved the study (YJSKY2025‐398). Both partners gave written informed consent and could omit questions or withdraw without affecting care. Individual responses remained confidential within dyads. Identifiers were removed, and electronic data were password‐protected, accessible only to authorized researchers, and retained and securely deleted under institutional requirements.

## 3 Results

### 3.1 Participant Characteristics

All 18 patient–spouse dyads completed interviews at all three time points. Patients were aged 28–59 years; 10 were male, 13 had ischemic stroke, and 12 had experienced a first‐ever stroke, while six had experienced recurrent stroke. Motor impairment was right‐sided in eight patients, left‐sided in eight, and bilateral in two. Spouses were aged 27–59 years; 10 were female, reported daily caregiving ranged from 2–4 to more than 12 h, and six had previous caregiving experience. Further participant characteristics are provided in Supplementary File 3.

### 3.2 Development of the Findings and the Patient–Spouse Dual‐Line Journey Map

Five cross‐cutting analytic domains were identified: (1) patients’ rehabilitation and everyday‐life experiences; (2) spouses’ caregiving experiences; (3) convergence and difference within dyads; (4) emotional experiences; and (5) stage‐specific support needs and service gaps. These domains identify the broad areas examined across participants and time points. The Results are organized chronologically by interview period, with findings from the five analytic domains presented within each period. This structure brings together patients’ and spouses’ experiences in the same care context and supports comparison of changes over time and differences within and between dyads. The three interview periods provide the temporal structure for reporting: T1: Inpatient recovery and preparation for discharge; T2: Early transition home and emerging differences in independence and safety; and T3: Longer‐term life reconstruction and varied dyadic adjustment. Presenting the findings chronologically makes visible how rehabilitation, caregiving, relationships, emotions, and support needs intersected as care moved from hospital to home.

Figure 1 brings these two dimensions together: rows identify the analytic domains, and columns identify the phase‐related findings using the same titles as the corresponding Results subsections. Supplementary File 4 links each domain and phase‐related finding to analytical categories and illustrative quotations. The emotional lines provide a schematic visual summary rather than numerical scores. The lighter branches at T3 indicate variation across dyads, including greater reassurance in some and continuing strain in others, rather than a fixed sequence or common endpoint.

**FIGURE 1:**
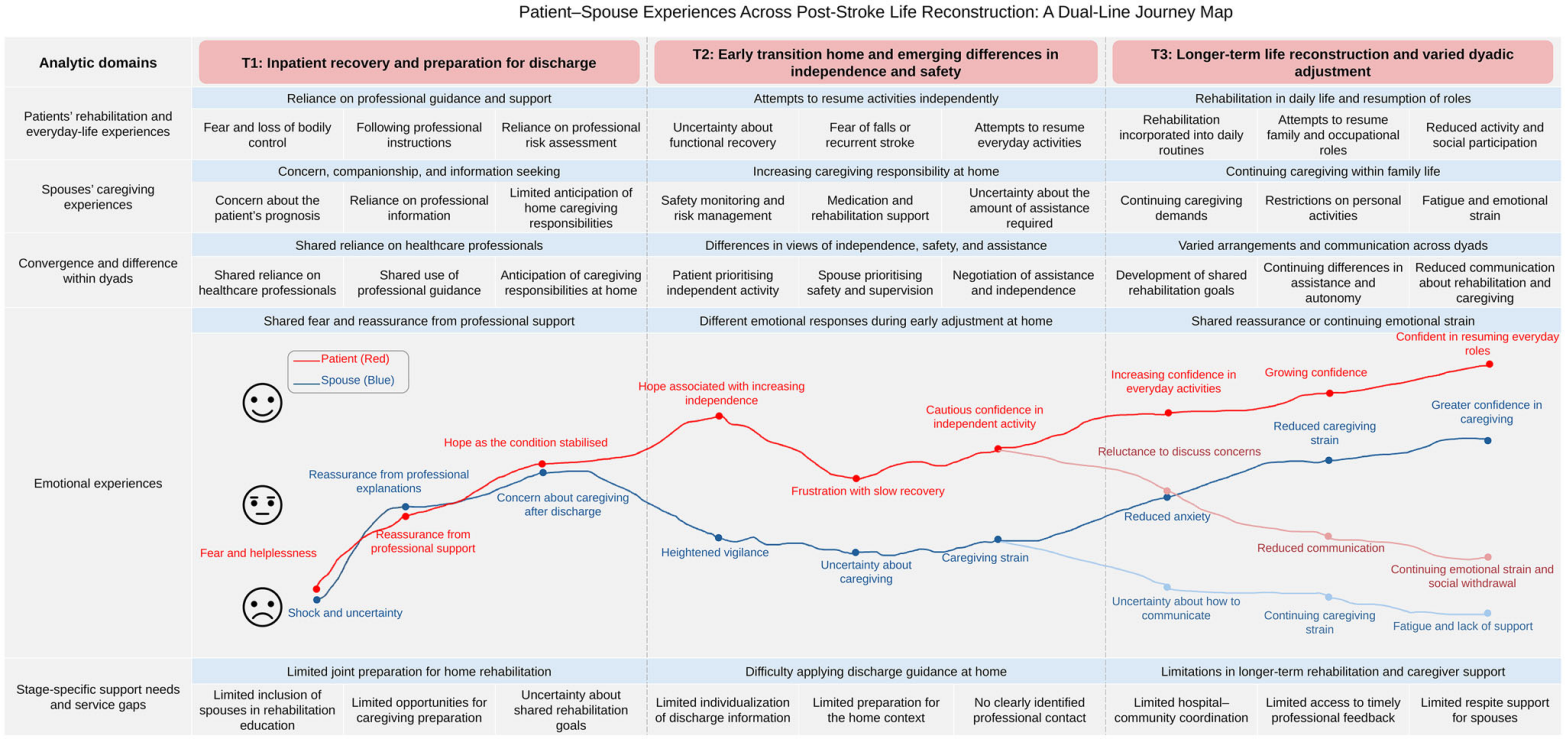
Patient–spouse dual‐line journey map integrating five analytic domains across three phase‐related findings after stroke. Note. Red and blue lines schematically represent patients’ and spouses’ emotional experiences, respectively; facial icons indicate broad emotional valence. Line positions are not quantitative. Lighter T3 branches represent variation across dyads rather than fixed outcomes or a universal recovery pathway.

### 3.3 Phase‐Related Findings Aligned With the Dual‐Line Journey Map

The following sections present the integrated phase‐related findings in chronological order. Within each phase, the account moves across the five analytic domains, describing patients’ rehabilitation and everyday life, spouses’ caregiving, convergence and difference within dyads, emotional experiences, and support needs. This structure preserves connections among the domains within the changing care context. Repeated accounts from the same participant are used to illustrate longitudinal change where available, while comparisons between dyads show variation without implying that all couples followed the same course.

#### 3.3.1 T1: Inpatient Recovery and Preparation for Discharge

During hospitalization, patients feared further harm when attempting movement, while spouses connected impaired mobility with uncertainty about family life. Within dyad 03, these concerns were expressed differently:

P03‐T1: “When it first happened, my arm and leg on this side just would not respond, and I was extremely frightened. When the nurse asked me to slowly lift my leg, I did not dare to use too much force because I was afraid that something would go wrong again if I moved.”

S03‐T1: “When I first learned that it was a stroke, my mind went blank. What I feared most at that time was that he might never be able to stand up again, and I did not know how our family would get by.”

Professional direction enabled participation without necessarily providing an understanding of rehabilitation or future caregiving. P11 described following instructions, whereas S12 retrospectively distinguished accompanying the patient in hospital from assuming responsibility at home:

P11‐T1: “At that time, I did not really understand what rehabilitation meant. I just did whatever the doctors and nurses told me to do. To be honest, I did not think too much about it; I just followed their instructions.”

S12: “In the hospital, there were doctors and nurses, so I mainly just stayed with him. At that time, I had not really realized that after going home, many things would fall on me.”

In dyad 06, both partners’ willingness to attempt movement depended on the nurse's presence. The patient sought reassurance about standing; the spouse sought reassurance about allowing the attempt:

P06‐T1: “I did not dare to get out of bed by myself because I was afraid my leg would suddenly become weak and I would fall. I only dared to try standing when the nurse was beside me.”

S06‐T1: “I became nervous whenever I saw him move, especially during the first few days. Only when the nurse was beside him did I dare to let him try.”

Shared fear did not always lead to shared discussion. Both members of dyad 03 concealed distress to avoid upsetting the other, leaving concerns privately held despite their mutual worry:

P03‐T1: “During the first few days after the onset, I was constantly panicked. I was afraid that I would remain like this in the future. When she came to see me, I did not dare to say too much because I was afraid, she would feel upset too.”

S03‐T1: “I was actually afraid, but I could not show it in front of him. Before going in to see him, I had to calm myself down first.”

Reassurance remained closely tied to the hospital environment. In dyad 10, routine checks reassured the patient, while immediate access to help reassured the spouse:

P10‐T1: “Although I was still afraid in the hospital, the doctors and nurses came to check on me every day, so I felt that someone was taking care of me, and I felt less uncertain.”

S10‐T1: “What I feared most at that time was that something might suddenly happen at night. Fortunately, we were in the hospital. When we pressed the call bell, someone would come, so I felt a little more reassured.”

However, feeling protected in hospital coexisted with incomplete preparation for home. Participants described uncertainty about both practical assistance and expected progress:

S12‐T1: “Before discharge, I also wanted to learn how to support him and how to help him exercise, but the time was too short. I felt that we went home before I had really learned it.”

P08‐T1: “The doctor said I should continue exercising at home, but neither of us had thought specifically about what level of recovery would count as good.”

Across these accounts, professional support made movement and caregiving feel more manageable within hospital, but reassurance did not necessarily translate into readiness for home. Patients’ uncertainty concerned what they could safely attempt and what progress to expect; spouses’ uncertainty also concerned how to provide help once professional supervision was no longer immediately available. Convergence at T1 therefore lay mainly in reliance on the clinical team, while practical responsibilities and shared rehabilitation goals remained incompletely worked out.

#### 3.3.2 T2: Early Transition Home and Emerging Differences in Independence and Safety

Four weeks after discharge, ordinary activities became opportunities to regain independence. For P05, doing an activity independently mattered more than speed; P14 simultaneously wanted independence and feared falling:

P05‐T2: “I now try to pour water by myself and walk a few steps while holding onto the table. It does not matter if I walk slowly; I just want to start doing what I can by myself.”

P14‐T2: “I want to walk to the living room by myself, but as soon as I take a step, I am afraid of falling. In the past, I never had to think so much about walking, but now I have to be careful with every step.”

Spouses described increased vigilance in the absence of immediately available professional help. Their uncertainty concerned both insufficient assistance and the possibility of encouraging dependence:

S05‐T2: “After we came home, I kept watching him all the time. I was afraid he would fall or that his blood pressure would become unstable. I felt even more nervous than in the hospital because at home there was no one who could deal with problems immediately.”

S02‐T2: “I do not know what I should help with and what I should not. If I help too much, I am afraid he will become dependent; if I do not help, I am afraid something will happen to him. It is really difficult to judge the right balance.”

Within dyad 05, understanding the patient's wish did not remove the spouse's fear. The patient emphasized the discomfort of dependence, whereas the spouse emphasized the consequences of a fall:

P05‐T2: “I do not want to depend on her for everything. If I can take a couple of steps by myself, I want to do it on my own. Always being supported by someone does not feel good.”

S05‐T2: “After coming home, he kept wanting to try things by himself. I can understand that, but I still feel scared when I watch him. Home is not like the hospital; if he falls, none of us can bear the consequences.”

Different assessments also reflected different reference points. P09 judged readiness against improvement since discharge; S09 judged safety against immediate hazards:

P09‐T2: “I feel that I am much better than when I was first discharged, so I want to move around more. But she always reminds me to slow down and not push myself too hard.”

S09‐T2: “He thinks he has recovered quite well, but I still do not feel confident. If there is a little water on the floor or if he walks a bit faster, I worry.”

Returning home could therefore carry different emotional meanings within the same couple. P02 linked movement with feeling like himself again, while S02 described being more anxious because responsibility now rested with the couple:

P02‐T2: “When I first returned home, I felt much stronger than when I was in the hospital, so I wanted to walk more. Staying in bed all the time was not a solution; the less I moved, the less I felt like myself.”

S02‐T2: “After coming home, I actually became more nervous. In the hospital, someone was watching over him, but at home it was just the two of us. Whenever he moved, I could not help staring at him because I was afraid he would fall or suddenly feel unwell.”

Longitudinal comparison of P11's accounts showed a change in how rehabilitation was evaluated. At T1, following professional instructions provided a way to participate despite limited understanding of rehabilitation. At T2, daily practice was assessed against visible improvement, and the perceived lack of progress became linked to frustration and a negative view of himself. S11's reassurance did not indicate an absence of concern: she described uncertainty while trying to contain her own distress in responding to his low mood. This differed from dyad 02, in which the patient's renewed activity carried a sense of hope alongside the spouse's increased vigilance:

P11‐T2: “I practice every day, but I cannot see much change. Sometimes I feel really annoyed and think, why am I so useless?”

S11‐T2: “When he is in a low mood, I also feel distressed, but I cannot become anxious with him. I can only tell him to take it slowly, although I actually feel uncertain myself.”

Participants also struggled to use discharge information when specific problems arose. P02 could not recall advice, while S16 was unsure whom to contact and reluctant to repeatedly call the ward. Managing alone reflected these difficulties rather than confidence:

P02‐T2: “A lot was said at discharge, but when we really encountered problems at home, I could not remember it, and I did not know whom to ask.”

S16‐T2: “After going home, we did not know whom to contact when we had questions. I also felt embarrassed to keep calling the ward, so we could only try to figure things out ourselves first.”

#### 3.3.3 T3: Longer‐Term Life Reconstruction and Varied Dyadic Adjustment

At six months, concern about dependence accompanied different patterns of participation. P15 described contributing through manageable household tasks and possible lighter employment. P17 described avoiding outings because of continuing impairment, social attention, and concern about burdening the spouse:

P15‐T3: “I help with some light housework whenever I can. I am also thinking about whether I can go back to doing some lighter work first. I cannot always have my family revolving around me.”

P17‐T3: “I have practiced for so long, but my walking is still unsteady. Sometimes I just do not want to go out. I am afraid others will notice, and I am also afraid of being a burden to her.”

For spouses, caregiving involved continuing attention as well as practical tasks. S11 remained preoccupied with the patient even when outside the home; S17 described realizing that daily responsibilities would extend beyond the initial period after discharge:

S11‐T3: “In the past, I could still go out to take care of my own things, but now I basically do not dare to go far. Even when I go out, I am always thinking about whether he can manage alone at home.”

S17‐T3: “Only after some time did, I realize that this was not just about the first few days after discharge. Every day, I have to think about his medication, training, meals, and walking. My mind is constantly tense.”

Dyadic arrangements varied. In dyad 04, smaller goals gave the patient clearer expectations, while the spouse described shifting from wanting rapid recovery to accepting a sustainable pace:

P04‐T3: “Now we discuss things together. For example, these days we first practice going downstairs, without rushing to walk too far. When the goal is smaller, I know what to expect.”

S04‐T3: “I used to always want him to recover quickly, but now I feel we cannot rush. We just take it step by step. Being able to keep going is more important than anything else.”

Both partners also associated the patient's increased independence with greater confidence or reduced anxiety:

P04‐T3: “Now I can do a few more things by myself, and I feel more confident. It feels like life is slowly getting back on track.”

S04‐T3: “Seeing that he can do some things by himself, I am not as panicked as before. Now I am not afraid of everything like I was at the beginning, and I feel a little more relaxed.”

In contrast, dyad 18 described progressively avoiding difficult topics. The patient intended silence to prevent additional worry, while the spouse withheld questions to avoid irritation. Less discussion thus coexisted with concerns that remained unspoken:

P18‐T3: “There are some things I do not want to say because if I say them, she will worry again. Sometimes I just keep them to myself because I do not want the atmosphere at home to become heavier.”

S18‐T3: “He does not talk much about things now, and if I ask too much, he gets annoyed. Gradually, there are some things we just do not mention anymore.”

In dyad 17, the patient's reluctance to discuss distress accompanied the restricted participation described earlier. The spouse felt caught between asking and risking annoyance, or remaining silent and leaving distress unaddressed:

P17‐T3: “Sometimes I do not want to trouble her, and I do not want to tell others either, so I just stay by myself. Talking too much does not solve anything and only makes my family worry.”

S17‐T3: “Sometimes he does not speak, and I do not know whether I should ask. If I ask too much, I am afraid he will become annoyed; if I do not ask, I am afraid he will keep everything bottled up.”

Continuing needs also concerned access to rehabilitation and alternative caregivers. P09, who had emphasized improvement and wanting more activity at T2, described difficulty finding rehabilitation at T3. S17 sought temporary relief from the daily responsibilities described above:

P09‐T3: “The community health service did not know about my situation either. I wanted to find a rehabilitation place, but I did not know where to go.”

S17‐T3: “I also wish someone could take over for me for a while, even just for half a day, but there is no one at home who can step in.”

At six months, life reconstruction involved both patients’ opportunities to resume valued activities and spouses’ experiences of sustaining care alongside their own lives. P15's efforts to contribute through household tasks and possible lighter employment differed from P17's restricted participation and reluctance to go out. Spouses’ accounts added the continuing demands of caregiving, including S11's restricted personal activities and S17's sustained vigilance and need for temporary relief. Within dyad 04, negotiated goals accompanied greater reassurance for both partners. Dyads 17 and 18 instead illustrated how concerns could remain unresolved as communication became difficult or reduced. The T3 findings therefore describe variation in participation, caregiving, emotional experiences, and dyadic adjustment, rather than a common endpoint reached by all couples.

## 4 Discussion

Repeated separate interviews showed that patients’ and spouses’ experiences changed as stroke care moved from hospital to home. During hospitalization, both partners relied on healthcare professionals for guidance and reassurance. After discharge, patients increasingly sought to resume activities independently, while spouses assumed greater responsibility for rehabilitation and safety. By six months, some dyads had negotiated acceptable goals and caregiving arrangements, whereas others continued to report restricted participation, emotional strain, or reduced communication. These findings describe variation across phases and dyads; they do not represent a universal recovery sequence.

### 4.1 Changing Responsibilities and Decision‐Making Across Care Settings

The transition home shifted many decisions about rehabilitation, assistance, and safety from professionals to families. Participants described difficulty applying general discharge advice to specific situations and uncertainty about whom to contact when questions arose. Ellis‐Hill et al. found that patients’ and carers’ experiences of discharge were shaped by whether they perceived continuing momentum in recovery and felt informed and supported (C. Ellis‐Hill et al. 2009). This helps interpret why returning home could carry different meanings within our dyads. Patients’ opportunities to resume familiar activities could coexist with spouses’ uncertainty about managing rehabilitation and safety without immediately available professional help. These observations are consistent with evidence of gaps in preparation, information, coordination, and timely support during stroke transitions (Chen et al. 2022; Chen et al. 2021; Lin et al. 2022; See Toh, Lim, Yobas, & Lim, 2022). Our findings show how such gaps were experienced within couples who shared recovery goals but could interpret progress, risk, and necessary assistance differently.

This pattern also aligns with a qualitative study in Ireland, where survivors and caregivers reported fragmented support, unmet emotional needs, and difficulty accessing information and services at the appropriate time (O'Callaghan et al. 2024). There are, however, important population differences. O'Callaghan et al. included survivors aged 47–79 and both spouse and adult‐child caregivers, recruited across different discharge pathways. Our study included married couples with patients aged 28–59 from one Chinese rehabilitation department. By interviewing both partners separately at three time points, we were able to examine how a shared service gap was experienced within a couple: patients’ attempts to resume activity could coincide with spouses’ increased safety monitoring. These findings suggest that uncertainty during transition is reported across settings, while the family responsibilities and life roles through which it is experienced may differ by population and context. They do not establish that the causes or organization of service gaps are the same across countries.

The Theory of Dyadic Illness Management helps interpret this interdependence: partners may share recovery goals while differing in their appraisals and strategies (Lyons and Lee 2018). In our study, differences did not invariably lead to conflict. By six months, some couples had developed shared goals and clearer boundaries, while others continued to experience uncertainty or communicated less. Support therefore needs to consider both partners’ perspectives and the circumstances of each dyad.

### 4.2 Independence, Identity, and Life Reconstruction in Young and Middle‐Aged Families

In this study, life reconstruction refers to participants’ efforts to sustain or renegotiate everyday activities, valued roles, and a sense of self while living with persistent impairment and changing caregiving arrangements. Bury's concept of biographical disruption highlights how illness can unsettle everyday assumptions, relationships, and expectations for the future (Bury 1982). Williams’ account of narrative reconstruction emphasizes how people make sense of illness by relating changed bodily and social circumstances to their life history (Williams 1984). These perspectives help explain why rehabilitation activities mattered to participants beyond their immediate physical demands.

Patients described feeling less like themselves when inactive, questioning their usefulness when recovery seemed slow, and wanting to contribute through household tasks or lighter employment. These accounts resonate with Ellis‐Hill and colleagues’ description of a separation between the familiar self and an altered, unreliable body following stroke (C. S. Ellis‐Hill et al. 2000). Satink and colleagues’ thematic synthesis similarly identified regaining valued roles and a sense of self as an ongoing struggle shaped by uncertainty and context (Satink et al. 2013). In our study, attempts to resume family and occupational contributions contrasted with restricted participation related to unsteady walking, concern about others’ attention, and fear of burdening a spouse. These differences suggest that life reconstruction involved adapting the meaning and performance of valued roles alongside continuing physical rehabilitation. They also complement evidence of unmet employment, family, and psychosocial needs among younger stroke survivors (Cleary et al. 2025).

The Life Thread Model draws attention to the interpersonal processes through which identity and continuity can be supported during rehabilitation (Ellis‐Hill Senior Lecturer et al. 2008). This relational perspective helps interpret the negotiation of independence and safety within our dyads. An activity that represented renewed competence or contribution to a patient could also represent additional risk and responsibility to a spouse. Such differences accord with dyadic research describing differing illness appraisals and management strategies after stroke (Wang et al. 2023). Some couples developed manageable shared goals, whereas others continued to experience restrictions or difficulty discussing concerns. Life reconstruction can therefore be understood as a negotiated process involving patients’ valued roles alongside spouses’ caregiving responsibilities and opportunities to maintain their own lives. These interpretations concern married couples with limb motor impairment in one Chinese rehabilitation setting; experiences in other family structures or among people with substantial cognitive or communication difficulties may differ.

### 4.3 Emotional Experiences and Communication Within Patient–Spouse Dyads

Both partners described fear and uncertainty, but their concerns were not identical. Patients emphasized function, dependence, and participation, whereas spouses emphasized safety, caregiving competence, and family demands. Quinn and colleagues’ meta‐synthesis identified adaptation to a changed role, putting personal needs aside, suffering in silence, and seeking space for personal well‐being and social support as important aspects of spousal coping after stroke (Quinn et al. 2014). These findings provide context for spouses’ restricted personal activities and requests for temporary relief in our study, and accord with evidence that caregiving can affect carers’ roles and valued activities (Jammal et al. 2024). Our paired accounts also showed that both partners could conceal distress to protect one another. Outward reassurance or limited disagreement could therefore coexist with privately held uncertainty. The contrasting accounts of negotiated goals and reduced communication indicate that emotional adjustment involved both individual coping and the ways concerns were expressed or withheld within the relationship.

These findings are consistent with research on linked psychological experiences and coping within stroke dyads (Lin et al. 2022; Song et al. 2025; Wang et al. 2023). They also resemble a longitudinal Swedish study in which spouses described changes in the marital relationship and emotional adjustment during the first year after discharge (Bäckström et al. 2010). The studies differ in who was interviewed and followed: Bäckström et al. followed four middle‐aged female spouses, whereas our study interviewed both partners in each dyad and included male and female spouses, with follow‐up through six months. These differences in sample composition and follow‐up period limit direct comparison, but the studies jointly highlight that relational and emotional needs can continue beyond discharge.

Separate opportunities to speak may help clinicians identify concerns that a partner is reluctant to share in a joint conversation. A joint discussion can then be offered when acceptable to both partners, to clarify expectations about activity, assistance, and caregiving boundaries. This is a practice implication drawn from participants’ accounts, rather than an intervention tested by the present study.

### 4.4 Stage‐Specific Implications for Transitional and Continuing Care

The findings point to different areas for discussion at different phases. Before discharge, both partners may benefit from practical preparation for activities they expect to manage at home, including an opportunity to demonstrate or practice relevant tasks and clarify which activities require assistance. Participants’ uncertainty about rehabilitation goals suggests agreeing on observable, manageable activities and how progress will be reviewed.

Early after discharge, the difficulties participants described in applying advice and finding professional help support a clear route for questions and follow‐up. A review of actual home routines could help identify whether the agreed level of assistance is workable and whether goals need adjustment. At six months, assessment could revisit patient participation and rehabilitation access alongside spouse workload, social participation, and caregiving strain. These phase‐related priorities are consistent with evidence on transitional care and caregiver preparation (Chen et al. 2021; O'Callaghan et al. 2024; See Toh et al. 2022).

These suggestions should be understood as priorities for individualized discussion, not as evidence that a particular follow‐up schedule is optimal. The journey map can help structure conversations about changing needs, but it has not been validated as a clinical assessment tool or as a predictor of an individual couple's trajectory.

### 4.5 Clinical Implications and the Limits of the Evidence

The recommendations in this study arise from reported needs: uncertainty about home rehabilitation, differing views of safety and assistance, concealed distress, and the demands of sustained caregiving. Existing guidance supports involving family caregivers in rehabilitation and providing education and training suited to their needs (Richards et al. 2026). Intervention research also offers some support for dyadic approaches. In a randomized controlled trial of 162 stroke survivor–caregiver dyads, Mou et al. found reductions in caregiver burden and improvements in caregiving competence and dyadic relationship outcomes following a family‐focused psychoeducational intervention; effects on survivor functioning were more limited (Mou, Lam, and Chien 2023). This evidence supports considering tailored dyadic support, while showing that benefits may vary by outcome.

In practice, rehabilitation teams could use discharge planning to discuss each partner's concerns, practice relevant activities, and document an individualized plan for assistance. Early follow‐up could review how the plan works at home and provide a clear contact for unresolved questions. Later reviews could consider participation, ongoing rehabilitation access, caregiver strain, and each partner's preferred involvement. For working‐age couples, discussion may also include employment, parenting, household responsibilities, and finances, which were salient in this sample and are reflected in research on younger stroke survivors (Cleary et al. 2025; Liu et al. 2025).

The present qualitative study did not test these practices or establish their effects. Their feasibility and acceptability, including whether they add to caregiver workload, should be evaluated before they are treated as effective interventions. Future studies could assess outcomes relevant to both partners, including participation, caregiver burden, communication, and satisfaction with agreed assistance.

### 4.6 Strengths and Limitations

Repeated separate interviews with both patients and spouses at three time points enabled longitudinal and within‐dyad comparison, with no loss to follow‐up. Longitudinal matrices, contrasting‐case analysis, and review by multiple researchers strengthened the credibility of the analysis. Nevertheless, participants came from one rehabilitation department in China and included married couples only; transferability to other healthcare settings, family structures, and caregiver relationships requires careful consideration.

Patients with substantial cognitive or communication difficulties were excluded, which may have limited the range of experiences represented. Self‐reported accounts may have been affected by social desirability or reluctance to disclose distress and disagreement. Separate interviews and reflexive memos helped address these influences but could not eliminate them. The study also did not test interventions or establish causal relationships between service access, couple communication, and recovery. Finally, the dual‐line journey map summarizes phase‐related variation and should not be interpreted as a universal recovery pathway or a measure of emotional change.

## 5 Conclusion

As care moved home, patients and spouses shared recovery goals but sometimes differed over independence, safety, and assistance. Six‐month arrangements ranged from mutually acceptable to persistently strained or poorly communicated. The dual‐line journey map summarizes these phase‐related experiences without implying a universal pathway. Findings support joint discharge preparation, individualized guidance and accessible early follow‐up, and stronger long‐term rehabilitation and caregiver support tailored to each dyad.

## Author Contributions

**Wenbo Li**: writing – original draft, visualization, methodology, formal analysis, data curation, conceptualization. **Qingfang Ye**: writing – original draft, methodology, formal analysis. **Haiyang Zhang**: writing – original draft, methodology, formal analysis. **Meng Wang**: methodology, formal analysis. **Yuying Fan**: writing – review and editing, visualization, supervision, software, project administration, methodology, formal analysis. **Qiujie Li**: writing – review and editing, visualization, supervision, software, project administration, methodology, formal analysis, conceptualization.

## Funding

This work was supported by the Heilongjiang Provincial Natural Science Foundation of China (BS2025G001).

## Ethics Statement

This study was conducted in accordance with the principles of the Declaration of Helsinki. Ethical approval was obtained from the Ethics Committee of Second Affiliated Hospital of Harbin Medical University (Approval No. YJSKY2025‐398). All procedures performed in studies involving human participants were in accordance with the ethical standards of the institutional and/or national research committee and with the 1964 Helsinki declaration and its later amendments or comparable ethical standards.

## Supporting information




**Table S1**. Consolidated criteria for reporting qualitative research (COREQ): 32‐item checklist

**Table S2‐A**. Longitudinal semi‐structured interview guide for young and middle‐aged stroke survivors with residual limb motor impairment

**Table S3**. Characteristics of the 18 patient–spouse dyads

**Table S4**. Phase‐related analytical categories and illustrative quotations supporting the patient–spouse dual‐line journey map

## Data Availability

The data that support the findings of this study are available from the corresponding author upon reasonable request.
