# Supplementary material for: Patient–Spouse Experiences of Post‐Stroke Life Reconstruction: A Longitudinal Dyadic Journey Mapping Study
Source: Brain Behav. 2026 Nov 9;16(11):e71914. doi: 10.1002/brb3.71914 (PMC13746992; doi:10.1002/brb3.71914)
Supplement: Supplementary file 1 [file BRB3-16-e71914-s001.docx]

**Table S1. Consolidated criteria for reporting qualitative research (COREQ): 32-item checklist**

| **No.** | **Item** | **Guide question/description** | **Reported in the manuscript** |
| --- | --- | --- | --- |
| **Domain 1: Research team and reflexivity** | | | |
| ***Personal characteristics*** | | | |
| 1 | Interviewer/facilitator | Which author(s) conducted the interview or focus group? | Page 8: The principal researcher conducted all interviews. |
| 2 | Credentials | What were the researcher’s credentials? | Page 7: The principal researcher was a doctoral nursing student. |
| 3 | Occupation | What was the researcher’s occupation at the time of the study? | Page 7: Not explicitly reported beyond doctoral study and involvement in clinical rehabilitation nursing practice. |
| 4 | Gender | Was the researcher male or female? | Not reported in the blinded manuscript. |
| 5 | Experience and training | What experience or training did the researcher have? | Page 7: Training in qualitative interviewing, research ethics, and reflexive practice; pilot interviews were supervised by senior researchers. |
| ***Relationship with participants*** | | | |
| 6 | Relationship established | Was a relationship established prior to study commencement? | Page 7: The principal researcher had prior contact with potential participants through clinical rehabilitation nursing practice but was not responsible for treating them. |
| 7 | Participant knowledge of the interviewer | What did participants know about the researcher? | Pages 7–8: Participants were informed of the researcher’s professional role, study purpose, reason for the research, voluntary participation, and confidentiality procedures. |
| 8 | Interviewer characteristics | What interviewer characteristics were reported, such as assumptions, interests, or possible bias? | Page 7: The researcher’s clinical background and possible influence were considered through reflexive memos throughout data collection and analysis. |
| **Domain 2: Study design** | | | |
| ***Theoretical framework*** | | | |
| 9 | Methodological orientation and theory | What methodological orientation was stated to underpin the study? | Pages 5 and 9–10: Framework Method adapted for longitudinal and dyadic qualitative data; the Theory of Dyadic Illness Management was used as a relational perspective in study design and interpretation. |
| ***Participant selection*** | | | |
| 10 | Sampling | How were participants selected? | Page 5: Maximum variation purposive sampling. |
| 11 | Method of approach | How were participants approached? | Pages 5–6: Potential participants were identified by the clinical team, eligibility was confirmed by the principal researcher, and verbal and written study information was provided. |
| 12 | Sample size | How many participants were in the study? | Page 11: 18 patient–spouse dyads (36 participants). |
| 13 | Non-participation | How many people refused to participate or dropped out, and for what reasons? | Page 11: No loss to follow-up among the 18 enrolled dyads. The numbers approached and declining participation, and reasons for refusal, were not reported. |
| ***Setting*** | | | |
| 14 | Setting of data collection | Where was the data collected? | Page 8: T1 interviews were conducted face-to-face in a quiet private room in the rehabilitation department; T2 and T3 were conducted in quiet private settings selected by participants. |
| 15 | Presence of non-participants | Was anyone else present besides participants and researchers? | Page 8: No family members or other individuals not involved in the research were present. |
| 16 | Description of sample | What are the important characteristics of the sample? | Page 11 and Supplementary File 3: Demographic, clinical, and caregiving characteristics. |
| ***Data collection*** | | | |
| 17 | Interview guide | Were questions, prompts, or guides by the authors? Was it pilot tested? | Page 8 and Supplementary File 2: Corresponding longitudinal guides for patients and spouses; pilot tested with five eligible individuals not included in the study. |
| 18 | Repeat interviews | Were repeat interviews carried out? | Pages 5 and 11: Each participant was interviewed at T1, T2, and T3, resulting in 108 interviews. |
| 19 | Audio/visual recording | Did the research use audio or visual recording? | Page 8: All interviews were audio-recorded with permission. |
| 20 | Field notes | Were field notes made during or after interviews? | Page 8: Field notes were made during or immediately after interviews; a reflexive memo was prepared after each interview. |
| 21 | Duration | What was the duration of interviews? | Page 8: Approximately 30–60 minutes. |
| 22 | Data saturation | Was data saturation discussed? | Page 6: Saturation was not claimed; sample adequacy was assessed using information power. |
| 23 | Transcripts returned | Were transcripts returned to participants for comment or correction? | Pages 8 and 10: Participants could correct or add to interview summaries; formal checking of completed findings was not claimed. |
| **Domain 3: Analysis and findings** | | | |
| ***Data analysis*** | | | |
| 24 | Number of data coders | How many data coders coded the data? | Pages 7 and 9–10: The principal researcher coded the complete dataset; three researchers reviewed selected coded extracts and matrices; two senior researchers supervised the analysis. |
| 25 | Description of the coding tree | Did the authors provide a description of the coding tree? | Pages 9–10 describe framework development and matrix analysis. Supplementary File 4 presents phase-related analytical categories and illustrative quotations rather than a formal coding tree. |
| 26 | Derivation of themes/categories | Were themes or categories identified in advance or derived from the data? | Pages 9–10: Coding was primarily data-driven. Interview-guide topics provided an initial organizational structure; new codes were added and the analytical framework was revised iteratively. |
| 27 | Software | What software, if applicable, was used to manage the data? | Not reported in the manuscript. |
| 28 | Participant checking | Did participants provide feedback on the findings? | Pages 8 and 10: Participants could correct or add to the interviewer’s summary during each interview. Formal participant checking of the completed findings was not claimed. |
| ***Reporting*** | | | |
| 29 | Quotations presented | Were participant quotations presented and identified? | Pages 12–19 and Supplementary File 4: Quotations are identified by patient/spouse, dyad number, and interview period. |
| 30 | Data and findings consistent | Was there consistency between the data presented and the findings? | Pages 11–19: Phase-related findings are supported by identified quotations and contrasting dyadic cases. |
| 31 | Clarity of major themes/categories | Were major themes or categories clearly presented? | Pages 11–12 and 29–30, Figure 1, and Supplementary File 4: Five analytical domains are presented across T1, T2, and T3. |
| 32 | Clarity of minor themes/diverse cases | Is there a description of diverse cases or discussion of minor themes? | Pages 12–19 and Supplementary File 4: Variation across dyads and contrasting T3 cases are presented. |
