# Supplementary material for: Patient–Spouse Experiences of Post‐Stroke Life Reconstruction: A Longitudinal Dyadic Journey Mapping Study
Source: Brain Behav. 2026 Nov 9;16(11):e71914. doi: 10.1002/brb3.71914 (PMC13746992; doi:10.1002/brb3.71914)
Supplement: Supplementary file 2 [file BRB3-16-e71914-s002.docx]

**Table S2-A. Longitudinal semi-structured interview guide for young and middle-aged stroke survivors with residual limb motor impairment**

| T1 (Inpatient Recovery Period) Interview Guide | |
| --- | --- |
| 1 | Please describe your main experiences during the inpatient recovery period after this stroke. What events or experiences impressed you the most? |
| 2 | How do you currently understand your physical recovery? What issues are you most concerned about at present? |
| 3 | During hospitalization, what kinds of help did healthcare professionals and your spouse provide? What impact did this help have on you? |
| 4 | As you are about to be discharged and return home, what thoughts or concerns do you have? |
| 5 | Have you and your spouse discussed post-discharge rehabilitation training, daily care, or family arrangements? How did you discuss these issues at that time? |
| 6 | What emotional changes have you experienced during this stage? Could you give some examples? |
| 7 | Before discharge, what issues do you feel remain unclear? What additional help would you like to receive? |
| T2 (Discharge Transition Period) Interview Guide | |
| 1 | After returning home, what changes have occurred in your daily life and rehabilitation training? |
| 2 | What activities are you now able to complete independently? What activities still require help from others? |
| 3 | Have you and your spouse had any disagreements regarding activities, training, medication, follow-up visits, or household tasks? How were these disagreements handled at the time? |
| 4 | After returning home, what attempts or changes have you made in terms of family life, work, or social life? How did this process go? |
| 5 | What emotional changes have you experienced during this stage? Which experiences impressed you the most? |
| 6 | Do you feel that post-discharge rehabilitation guidance, follow-up care, community-based rehabilitation, or family support has met your needs? What gaps or shortcomings remain? |
| 7 | If healthcare professionals could continue to provide support, in which areas would you most like to receive help? |
| T3 (Home-Based Life Reconstruction Period) Interview Guide | |
| 1 | Looking back on the period since discharge, what changes do you feel have occurred in your life? |
| 2 | How are rehabilitation training, daily activities, and family life currently arranged? |
| 3 | How do you and your spouse currently arrange rehabilitation, daily care, and family affairs? How do you view these arrangements? |
| 4 | What impact has this experience had on communication and interactions between you and your spouse? |
| 5 | How does your current emotional state compare with when you were first discharged? What are your thoughts about future recovery and life? |
| 6 | Looking back on the entire process of rebuilding life after discharge, which stage was the most difficult for you? Why? |
| 7 | At which time points do you think support from hospitals, communities, or nursing staff would be more useful? What specific help would you like to receive? |

**Table S2-B. Longitudinal semi-structured interview guide for spouses of young and middle-aged stroke survivors with residual limb motor impairment**

| T1 (Inpatient Recovery Period) Interview Guide | |
| --- | --- |
| 1 | Please describe your main experiences of accompanying and caring for the patient during the inpatient recovery period. What events or experiences impressed you the most? |
| 2 | How do you currently understand the patient’s recovery? What issues are you most concerned about at present? |
| 3 | During hospitalization, what did you mainly do for the patient? How did you view your role at that time? |
| 4 | As the patient is about to be discharged, what preparations have you made? In which areas do you still feel uncertain or unprepared? |
| 5 | Have you and the patient discussed post-discharge rehabilitation training, care arrangements, or the division of family responsibilities? How did you discuss these issues at that time? |
| 6 | What emotional changes have you experienced during this stage? Could you give some examples? |
| 7 | Before discharge, do you feel that the guidance provided by healthcare professionals for spouse caregivers met your needs? What specific guidance would you still like to receive? |
| T2 (Discharge Transition Period) Interview Guide | |
| 1 | After the patient returned home, what changes occurred in your caregiving tasks? |
| 2 | What do you mainly help the patient with each day? Which tasks have had a greater impact on you? |
| 3 | Have you and the patient had any disagreements regarding the patient’s activities, training, medication, follow-up visits, or division of household tasks? How were these disagreements handled at the time? |
| 4 | How do you usually judge whether the patient can safely complete certain activities? |
| 5 | What impact has caregiving had on your work, family responsibilities, financial situation, or personal life? |
| 6 | What emotional changes have you experienced during this stage? Which caregiving experiences impressed you the most? |
| 7 | Do you feel that post-discharge follow-up, community-based rehabilitation, caregiving guidance, or family support has met your needs? What gaps or shortcomings remain? |
| T3 (Home-Based Life Reconstruction Period) Interview Guide | |
| 1 | Looking back on this caregiving experience, how has your caregiving role changed? |
| 2 | At present, how are the patient’s rehabilitation, daily life, and family affairs mainly arranged? |
| 3 | How do you and the patient currently discuss rehabilitation goals, life arrangements, and family responsibilities? What impact do you think this approach has on your current life? |
| 4 | What impact has this experience had on the marital relationship between you and the patient? |
| 5 | Looking back on this caregiving process, what emotional changes have you experienced? Which experiences impressed you the most? |
| 6 | Looking back on the entire process, which stage was the most difficult for you? Why? |
| 7 | What kinds of support do you think spouse caregivers need most during the process of rebuilding life after discharge? |

**Note.** The guides were used flexibly. Question order and wording were adapted to participants’ responses, and relevant issues raised in earlier interviews were revisited at T2 and T3 when clarification or further exploration was required.
