# Supplementary material for: Patient–Spouse Experiences of Post‐Stroke Life Reconstruction: A Longitudinal Dyadic Journey Mapping Study
Source: Brain Behav. 2026 Nov 9;16(11):e71914. doi: 10.1002/brb3.71914 (PMC13746992; doi:10.1002/brb3.71914)
Supplement: Supplementary file 3 [file BRB3-16-e71914-s003.docx]

**Table S3. Characteristics of the 18 patient–spouse dyads**

| **Dyad ID** | **Patient characteristics** | | | | | | | | **Spouse characteristics** | | | | | | |
| --- | --- | --- | --- | --- | --- | --- | --- | --- | --- | --- | --- | --- | --- | --- | --- |
|  | **Sex** | **Age** | **Educational attainment** | **Employment status** | **Place of residence** | **Stroke type** | **First-ever stroke** | **Side of limb motor impairment** | **Sex** | **Age** | **Educational attainment** | **Employment status** | **Self-reported health status** | **Reported average daily caregiving time** | **Previous caregiving experience** |
| P01/S01 | M | 28 | Bachelor degree | Employed | Urban | Ischemic | Yes | Right | F | 27 | Bachelor degree | Employed | Good | 2–4 h/day | No |
| P02/S02 | F | 32 | College | Employed | Urban | Ischemic | Yes | Left | M | 34 | College | Employed | Good | 3–5 h/day | No |
| P03/S03 | M | 36 | Senior high school | Employed | County town | Hemorrhagic | Yes | Right | F | 35 | Senior high school | Employed | Good | 4–6 h/day | No |
| P04/S04 | F | 39 | Bachelor degree | Employed | Urban | Ischemic | Yes | Left | M | 40 | Bachelor degree | Employed | Good | 3–5 h/day | No |
| P05/S05 | M | 41 | Junior high school | Manual worker | Rural | Ischemic | No | Right | F | 39 | Junior high school | Homemaker | Fair | 8–10 h/day | Yes |
| P06/S06 | M | 43 | College | Self-employed | Urban | Hemorrhagic | Yes | Left | F | 42 | College | Self-employed | Good | 6–8 h/day | No |
| P07/S07 | F | 45 | Senior high school | Employed | County town | Ischemic | Yes | Right | M | 47 | Senior high school | Employed | Good | 4–6 h/day | No |
| P08/S08 | M | 47 | Bachelor degree | Employed | Urban | Ischemic | No | Left | F | 46 | College | Employed | Fair | 6–8 h/day | Yes |
| P09/S09 | F | 49 | Primary school | Farmer | Rural | Ischemic | Yes | Right | M | 51 | Junior high school | Farmer | Fair | 8–10 h/day | No |
| P10/S10 | M | 50 | Senior high school | Sick leave | Urban | Hemorrhagic | Yes | Left | F | 48 | Senior high school | Employed | Good | 8–10 h/day | No |
| P11/S11 | M | 52 | Junior high school | Unemployed | Rural | Ischemic | No | Right | F | 50 | Primary school | Homemaker | Fair | 10–12 h/day | Yes |
| P12/S12 | F | 53 | College | Flexible employment | Urban | Ischemic | Yes | Left | M | 55 | Senior high school | Flexible employment | Fair | 6–8 h/day | No |
| P13/S13 | M | 54 | Primary school | Manual worker | Rural | Hemorrhagic | No | Bilateral | F | 52 | Primary school | Farmer | Fair | >12 h/day | Yes |
| P14/S14 | F | 55 | Junior high school | Homemaker | County town | Ischemic | Yes | Right | M | 57 | Junior high school | Retired | Fair | 8–10 h/day | No |
| P15/S15 | M | 56 | College | Employed | Urban | Ischemic | No | Left | F | 54 | College | Employed | Good | 4–6 h/day | Yes |
| P16/S16 | F | 57 | Senior high school | Sick leave | Rural | Hemorrhagic | Yes | Right | M | 58 | Senior high school | Farmer | Fair | 10–12 h/day | No |
| P17/S17 | M | 58 | Junior high school | Retired early | County town | Ischemic | No | Bilateral | F | 56 | Junior high school | Homemaker | Poor | >12 h/day | Yes |
| P18/S18 | F | 59 | Bachelor degree | Employed | Urban | Ischemic | Yes | Left | M | 59 | Bachelor degree | Employed | Good | 4–6 h/day | No |

**Note.** P = patient; S = spouse; M = male; F = female. Patient and spouse identifiers sharing the same number belong to the same dyad. Caregiving time and spouse health status were self-reported.
