# Supplementary material for: Patient–Spouse Experiences of Post‐Stroke Life Reconstruction: A Longitudinal Dyadic Journey Mapping Study
Source: Brain Behav. 2026 Nov 9;16(11):e71914. doi: 10.1002/brb3.71914 (PMC13746992; doi:10.1002/brb3.71914)
Supplement: Supplementary file 4 [file BRB3-16-e71914-s004.docx]

**Table S4. Phase-related analytical categories and illustrative quotations supporting the patient–spouse dual-line journey map**

| **Analytic domain** | **Phase-related finding** | **Phase-related analytical category** | **Illustrative quotations** |
| --- | --- | --- | --- |
| **Patients’ rehabilitation and everyday-life experiences** | **T1: Inpatient recovery and preparation for discharge** | **Reliance on professional guidance and support** | **P03-T1:** “When it first happened, my arm and leg on this side just would not respond, and I was extremely frightened. When the nurse asked me to slowly lift my leg, I did not dare to use too much force because I was afraid that something would go wrong again if I moved.”  **P11-T1:** “At that time, I did not really understand what rehabilitation meant. I just did whatever the doctors and nurses told me to do. To be honest, I did not think too much about it; I just followed their instructions.” |
|  | **T2: Early transition home and emerging differences in independence and safety** | **Attempts to resume activities independently** | **P05-T2:** “I now try to pour water by myself and walk a few steps while holding onto the table. It does not matter if I walk slowly; I just want to start doing what I can by myself.”  **P14-T2:** “I want to walk to the living room by myself, but as soon as I take a step, I am afraid of falling. In the past, I never had to think so much about walking, but now I have to be careful with every step.” |
|  | **T3: Longer-term life reconstruction and varied dyadic adjustment** | **Rehabilitation in daily life and resumption of roles** | **P15-T3:** “I help with some light housework whenever I can. I am also thinking about whether I can go back to doing some lighter work first. I cannot always have my family revolving around me.”  **P17-T3:** “I have practiced for so long, but my walking is still unsteady. Sometimes I just do not want to go out. I am afraid others will notice, and I am also afraid of being a burden to her.” |
| **Spouses’ caregiving experiences** | **T1: Inpatient recovery and preparation for discharge** | **Concern, companionship, and information seeking** | **S03-T1:** “When I first learned that it was a stroke, my mind went blank. What I feared most at that time was that he might never be able to stand up again, and I did not know how our family would get by.”  **S12-T1:** “In the hospital, there were doctors and nurses, so I mainly just stayed with him. At that time, I had not really realized that after going home, many things would fall on me.” |
|  | **T2: Early transition home and emerging differences in independence and safety** | **Increasing caregiving responsibility at home** | **S05-T2:** “After we came home, I kept watching him all the time. I was afraid he would fall or that his blood pressure would become unstable. I felt even more nervous than in the hospital because at home there was no one who could deal with problems immediately.”  **S02-T2:** “I do not know what I should help with and what I should not. If I help too much, I am afraid he will become dependent; if I do not help, I am afraid something will happen to him. It is really difficult to judge the right balance.” |
|  | **T3: Longer-term life reconstruction and varied dyadic adjustment** | **Continuing caregiving within family life** | **S11-T3:** “In the past, I could still go out to take care of my own things, but now I basically do not dare to go far. Even when I go out, I am always thinking about whether he can manage alone at home.”  **S17-T3:** “Only after some time did I realize that this was not just about the first few days after discharge. Every day, I have to think about his medication, training, meals, and walking. My mind is constantly tense.” |
| **Convergence and difference within dyads** | **T1: Inpatient recovery and preparation for discharge** | **Shared reliance on healthcare professionals** | **P01-T1:** “When I was first hospitalized, I knew nothing. I just practiced according to what the doctors and nurses told me, and that made me feel a little more reassured.”  **S01-T1:** “At that time, in the hospital, I mainly followed the arrangements made by the doctors and nurses. As long as they said his condition was stable, I felt less panicked.” |
|  | **T2: Early transition home and emerging differences in independence and safety** | **Differences in views of independence, safety, and assistance** | **P05-T2:** “I do not want to depend on her for everything. If I can take a couple of steps by myself, I want to do it on my own. Always being supported by someone does not feel good.”  **S05-T2:** “After coming home, he kept wanting to try things by himself. I can understand that, but I still feel scared when I watch him. Home is not like the hospital; if he falls, none of us can bear the consequences.” |
|  | **T3: Longer-term life reconstruction and varied dyadic adjustment** | **Varied arrangements and communication across dyads** | **P04-T3:** “Now we discuss things together. For example, these days we first practice going downstairs, without rushing to walk too far. When the goal is smaller, I know what to expect.”  **S04-T3:** “I used to always want him to recover quickly, but now I feel we cannot rush. We just take it step by step. Being able to keep going is more important than anything else.”  **P18-T3:** “There are some things I do not want to say because if I say them, she will worry again. Sometimes I just keep them to myself because I do not want the atmosphere at home to become heavier.”  **S18-T3:** “He does not talk much about things now, and if I ask too much, he gets annoyed. Gradually, there are some things we just do not mention anymore.” |
| **Emotional experiences** | **T1: Inpatient recovery and preparation for discharge** | **Shared fear and reassurance from professional support** | **P03-T1:** “During the first few days after the onset, I was constantly panicked. I was afraid that I would remain like this in the future. When she came to see me, I did not dare to say too much because I was afraid she would feel upset too.”  **S03-T1:** “I was actually afraid, but I could not show it in front of him. Before going in to see him, I had to calm myself down first.” |
|  | **T2: Early transition home and emerging differences in independence and safety** | **Different emotional responses during early adjustment at home** | **P02-T2:** “When I first returned home, I felt much stronger than when I was in the hospital, so I wanted to walk more. Staying in bed all the time was not a solution; the less I moved, the less I felt like myself.”  **S02-T2:** “After coming home, I actually became more nervous. In the hospital, someone was watching over him, but at home it was just the two of us. Whenever he moved, I could not help staring at him because I was afraid he would fall or suddenly feel unwell.”  **P11-T2:** “I practice every day, but I cannot see much change. Sometimes I feel really annoyed and think, why am I so useless?”  **S11-T2:** “When he is in a low mood, I also feel distressed, but I cannot become anxious with him. I can only tell him to take it slowly, although I actually feel uncertain myself.” |
|  | **T3: Longer-term life reconstruction and varied dyadic adjustment** | **Shared reassurance or continuing emotional strain** | **P04-T3:** “Now I can do a few more things by myself, and I feel more confident. It feels like life is slowly getting back on track.”  **S04-T3:** “Seeing that he can do some things by himself, I am not as panicked as before. Now I am not afraid of everything like I was at the beginning, and I feel a little more relaxed.”  **P17-T3:** “Sometimes I do not want to trouble her, and I do not want to tell others either, so I just stay by myself. Talking too much does not solve anything and only makes my family worry.”  **S17-T3:** “Sometimes he does not speak, and I do not know whether I should ask. If I ask too much, I am afraid he will become annoyed; if I do not ask, I am afraid he will keep everything bottled up.” |
| **Stage-specific support needs and service gaps** | **T1: Inpatient recovery and preparation for discharge** | **Limited joint preparation for home rehabilitation** | **S12-T1:** “Before discharge, I also wanted to learn how to support him and how to help him exercise, but the time was too short. I felt that we went home before I had really learned it.”  **P08-T1:** “The doctor said I should continue exercising at home, but neither of us had thought specifically about what level of recovery would count as good.” |
|  | **T2: Early transition home and emerging differences in independence and safety** | **Difficulty applying discharge guidance at home** | **P02-T2:** “A lot was said at discharge, but when we really encountered problems at home, I could not remember it, and I did not know whom to ask.”  **S16-T2:** “After going home, we did not know whom to contact when we had questions. I also felt embarrassed to keep calling the ward, so we could only try to figure things out ourselves first.” |
|  | **T3: Longer-term life reconstruction and varied dyadic adjustment** | **Limitations in longer-term rehabilitation and caregiver support** | **P09-T3:** “The community health service did not know about my situation either. I wanted to find a rehabilitation place, but I did not know where to go.”  **S17-T3:** “I also wish someone could take over for me for a while, even just for half a day, but there is no one at home who can step in.” |

**Note.** P = patient; S = spouse. Patient and spouse identifiers sharing the same number belong to the same dyad. T1 = 1–3 days before discharge; T2 = 4 weeks after discharge; T3 = 6 months after discharge. Quotations are illustrative rather than exhaustive. The categories summarise phase-related patterns identified through longitudinal, within-dyad, and between-dyad comparisons and should not be interpreted as a fixed sequence followed by all dyads.
